# Farm-Level Risk Factors for Lameness in 659 German Dairy Herds Kept in Loose Housing Systems

**DOI:** 10.3390/ani14172578

**Published:** 2024-09-04

**Authors:** Anna Tillack, Roswitha Merle, Kerstin-Elisabeth Müller, Martina Hoedemaker, Katharina Charlotte Jensen, Andreas W. Oehm, Marcus Klawitter, Annegret Stock

**Affiliations:** 1 Farm Animal Clinic, Division for Ruminants and Camelids, Unit for Internal Medicine and Surgery, School of Veterinary Medicine, Freie Universität Berlin, 14163 Berlin, Germany; 2 School of Veterinary Medicine, Institute of Veterinary Epidemiology and Biostatistics, Freie Universität Berlin, 14163 Berlin, Germany; 3 Clinic for Cattle, Clinical Centre for Farm Animals, University of Veterinary Medicine Foundation, 30173 Hannover, Germany; 4 Clinic for Ruminants with Ambulatory and Herd Health Services, Ludwig-Maximilians University Munich, 85764 Munich, Germany; 5 Zoetis Deutschland GmbH, 10785 Berlin, Germany

**Keywords:** lameness prevalence, dairy cows, cubicle design, feeding management, lameness assessment

## Abstract

**Simple Summary:**

Lameness is an important welfare issue in dairy farming that is causing substantial economic losses. This study aims to determine the association of potential risk factors with farm-level lameness in German dairy herds, including cubicle design and cubicle bedding, feeding management, lameness assessment, claw health management, stocking density, and floor design. Risk factors were identified for all cows regardless of the number of calvings (primiparous and multiparous cows) and for first lactation cows separately. Results of the present study showed that larger cubicle width and deep bedded cubicles are associated with a lower risk of being lame. In farms feeding a total mixed ration, the risk of being lame was lower than in farms with other feeding routines (partial mixed ration or single components). For first lactation cows, the way and frequency of lameness assessment were associated with lameness. Many of the factors revealed by this study are related to cow comfort, especially the comfort when lying down. More attention is needed here to reduce lameness in German dairy cows.

**Abstract:**

Six hundred fifty-nine farms in three regions of Germany (North: *n* = 240, East: *n* = 247, and South: *n* = 172) were included in the study, which aims at determining the association of management-related risk factors with farm-level lameness in German dairy herds. For each risk factor, a generalised linear regression model with negative binomial distribution and logit link was built. Results showed that cows housed in deep-bedded cubicles had a lower risk of being lame than cows housed in other cubicle types. A larger cubicle width was associated with a lower risk of being lame. Feeding a total mixed ration was associated with lower lameness prevalence (compared to feeding a partial mixed ration or single components). For first lactation cows, lameness assessment performed daily (compared to less than daily) and during other work tasks (compared to lameness assessment as a separate work task) were associated with lower risk for lameness. Finally, the present study provided evidence for crucial associations of management-related risk factors with lameness in German dairy cows, especially in the fields of cubicle design, feeding management, and lameness assessment.

## 1. Introduction

Lameness is one of the major issues in the dairy industry worldwide, impairing the welfare of cows [1] and creating huge economic losses [2,3,4,5]. Lameness prevalence in dairy cows varies between countries. The mean percentage reported for lameness prevalence in German dairy herds varies between 15.7% [6] and 29.4% [7]; for other Western countries similar prevalences were determined [8,9,10,11]. Differences in lameness prevalence on dairy farms are attributable to differences in animal husbandry and management systems. The high percentage of lame cows on dairy farms in Germany is alarming and requires interventions that are suitable to decrease the prevalence of lameness. Lameness in dairy cows is a condition multifactorial by origin, reflecting the incapability of the animals to cope with the environmental and management conditions they are exposed to. Important animal-based factors contributing to an increased risk of lameness are age, milk yield, days in milk and body condition [12,13,14,15,16]. Crucial factors contributing to the development of claw lesions and, thus, lameness are stall design, floor and cubicle hygiene, and claw health management. Numerous studies have shown the protective effect with respect to lameness following improvements in housing conditions and management [17,18,19,20,21]. Major deficiencies were identified by the latter authors with respect to the following items: slippery flooring, inadequate bedding and dimensions of cubicles, high stocking densities, and frequency of preventive claw trimming. Furthermore, it seems that herds that are housed permanently in free stalls with cubicles show a higher prevalence of lameness than pasture-based herds or cows that are housed in tie stalls [22,23]. The composition of the ration, as well as the feeding technology, have been discussed in previous studies as risk factors for lameness [24,25,26]. Rations with low fibre and high starch proportions can lead to rumen acidosis and, subsequently, to laminitis [27]. Even though numerous studies focused on risk factors, only a limited number of farms have been included in these studies. Moreover, most studies examining risk factors for lameness were conducted in countries other than Germany that might differ in management.

Therefore, the aim of this study was to determine risk factors on the farm level in German dairy herds with loose housing systems. The data was collected within a large-scale research study. Previous publications of this project focused on risk factors for tie stall-housed cows [28], the association between lameness prevalence and access to pasture [7] and cow-level risk factors such as body condition and lactation stage [29].

We hypothesised that cubicle design, feeding management, lameness assessment, claw health management, stocking density, and floor design are associated with lameness prevalence, and there are differences between first lactation cows and the entire herd.

## 2. Materials and Methods

This study was part of a larger consortium project named ‘PraeRi’ [30], funded by the German Federal Ministry of Food and Agriculture through the Federal Office of Agriculture and Food. In this cross-sectional observational study, data on housing conditions, health and biosecurity on German dairy farms was collected; further details of the study design are described by Jensen et al. [31] and Merle et al. [32]. The aims of the PraeRi study were (1) a representative description of dairy cow health under the current conditions in Germany and (2) the development of options for action for all groups working on dairy farms (farmer, veterinarian, hoof trimmer, other advisors). These were meant to serve as a basis for further investigations and political discussions.

### 2.1. Farm Recruitment and Data Collection

A total of 765 farms was enrolled in the PraeRi study, distributed over three regions: North (N) with the federal states Schleswig-Holstein and Lower Saxony, East (E) with the federal states Mecklenburg-West Pomerania, Brandenburg, Thuringia and Saxony-Anhalt, and South (S) with the federal state Bavaria.

In each region, a sample of dairy farms (N: *n* = 2787, E: *n* = 1739, S: *n* = 4418) was randomly selected based on the national animal information database (HI-Tier, München, Germany), on the farm data from the Milchpruefring Bayern e.V. (Wolnzach, Germany) in S and from the national milk recording associations (LKV) in N and E. A detailed sampling procedure is presented by Rittweg et al. [29]. Three herd size categories were generated for every region, i.e., small, medium, and large, depending on the number of milking cows (Table 1).

For each category, a separate sample size was calculated as described by Rittweg et al. [29]. Farmers were asked by postcard for voluntary participation. After receiving reply postcards or emails, the farmers were contacted by phone to check if the farming system fulfilled the recruitment criterion: the housing of dairy cows for commercial reasons (sale of milk). All farms fulfilling the criterion (N: *n* = 253, E: *n* = 252, S: *n* = 260) were visited once by a team of trained veterinarians. The preparation and training of observers (*n* = 22) consisted of a workshop including group discussions and video sessions, as well as continuous telephone conferences. In addition to that, all observers completed a pilot phase of three months before the actual start of the PraeRi study to get acquainted with the procedures, e.g., lameness assessment, during the farm visits. During the study period, which lasted from December 2016 until August 2019, three seminars took place in which inter-observer reliability was assessed, and group discussions, practical courses, and video sessions took place.

During the farm visit, animal-, management-, and housing-based data were collected by interviews, observations, and measurements. Furthermore, herd health and performance records for the twelve months before the visit were backed up. Parity and breed of cows were collected from the national milk recording associations of each region (Waldsieversdorf, Halle, Erfurt, Güstrow, Leer, and Kiel, Germany). Herd size was collected from the HI-Tier (München, Germany). Information on farming type (conventional or organic), milking system, feeding management and feed composition, claw trimming routines, culling rate due to lameness and access to pasture or exercise areas were obtained in interviews with the herd managers or farm owners. After the interview, the farmers completed an adapted HEXACO questionnaire, a validated model to assess human personality traits [33]. This was performed in private to ensure confidentiality requirements, and further information concerning the questionnaire can be retrieved from Adler and Campe [34].

Lameness was assessed using a modified 5-point-scale visual locomotion scoring by Sprecher et al. [35]; modifications were described by Tillack et al. [7]. Locomotion scoring was performed on lactating and dry cows while they were in their groups and were able to move freely in their free stalls, straw yards or on pasture. Standard operating procedures were provided, and the observers were trained in inter-observer assessments once a year (overall three times). The interobserver agreement (weighted kappa) varied between 0.39 and 0.63 [29], which indicates a moderate agreement [36]. For further analysis, locomotion scores (LS) from the Sprecher score were dichotomised into non-lame (LS < 3) and lame (LS ≥ 3).

On each farm, data on flooring type, manure removing system, cubicle dimensions, cubicle design, and bedding type in all compartments with dairy cows was recorded in standardised protocols. These protocols were developed based on the literature and expert opinions. Repeatability, validity, and feasibility were considered. The protocols determined which method should be used to obtain information, at what level (farm, compartment, cow level) and for which group (e.g., first lactation, dry cows) information should be collected [30]. The following dimensions of the cubicles were recorded: width, neck-rail-to-curb-distance, brisket board height and curb height. This is also explained in detail in Freigang et al. [37]. The exact number of cubicles that had to be measured was calculated depending on farm size prior to the farm visit: <30 cubicles per farm: 10 cubicles were measured; 30–49 cubicles: 15 cubicles; 50–99 cubicles: 17 cubicles; ≥100 cubicles: 18 cubicles. Of all cubicle parameters, the median per farm was calculated for further analyses. The number of cows, cubicles (one cubicle equals 4.0 m^2^ in free stalls without cubicles) and feeding places (one headlock equals 0.75 m feed bunk space) per compartment were recorded to obtain the animal-feeding place ratio (AFR) and animal-stall ratio (ASR) for each compartment.

The roughage ratio (in % per kg dry matter) in the ration of lactating/high-yielding cows was calculated with the program Futter R version 5 (dsp agrosoft, Ketzin/Havel, Germany) using the information about feed composition obtained in the interview and by using the results of feed analysis of all primarily used silages and hay.

### 2.2. Data Management and Statistical Analyses

Data were stored in a relational SQL database; relevant tables were converted into CSV format and imported into the statistic program SAS 9.4 (SAS Institute Inc. 2013, Cary, NC, USA) for further analyses. The data were checked for plausibility and incomplete or incorrect data; if necessary, observations were excluded from further analyses. This was also the case for farms with more than 10% of cows kept in tie-stalls on the day of the farm visit. Results of risk factor analyses for lameness in tie-stalls can be retrieved from the study of Oehm et al. [28].

All statistical analyses in the current study were performed at the farm level. Since data collection was performed on cow, compartment, and farm levels, some of the obtained variables had to be collated to the farm level. Categorical variables on the compartment level were modified to modal values per farm, and continuous variables were aggregated to median values per farm (Table 2 and Table 3).

Furthermore, some variables were newly generated and/or categorised. This was the case for the “predominant housing system”, with the following categories: “loose housing with cubicles”, “straw-based free stalls”, “pasture-based”, and “mixed”. If more than 80% of the cows were kept in the respective housing system on the day of the farm visit, the farm was assigned to the appropriate group. If several different systems were present, but none of them for more than 80% of the cows on the farm, the farm was assigned to the group “mixed”. The breed was classified accordingly (>80% of cows at farm visit) into the following four categories: “Holstein-Friesian”, “Simmental”, “Brown Swiss”, and “others” (including farms with no predominant breed). AFR and ASR were surveyed per compartment, and the mean values were calculated per farm weighted by the number of cows per compartment. ASR, as opposed to AFR, was categorised into three groups, i.e., “poor” (>1.2), “moderate” (1–1.2), and “good” (≤1:1), because it was not normally distributed. The variable “predominant cubicle type” implies that more than 80% of the cows on the day of the farm visit were kept in a compartment with the according cubicle type, i.e., “raised deep stall”, “raised stall”, “deep stall”, or “others”. Information on cubicle type and the existence of a bedding material were combined into the new variable “cubicle” with the categories “raised stall with bedding”, “raised stall without bedding”, and “deep stall” (by definition with bedding).

The frequency of feed submission and ration type were documented for each ration of the dairy cows. For further analysis, the mean frequency of feed submission for lactating cows over all rations was categorised because it was not normally distributed. The most common (modal value) ration type of all dairy cows (lactating and dry) in each farm was used for the analysis.

For each risk factor, a causal directed acyclic graph (DAG) was constructed using DAGITTY (www.dagitty.net, accessed on 24 April 2023) to identify causal confounders [38]. For each DAG, a generalised linear regression model with negative binomial distribution and logit link was built (a) only including the risk factor (univariable model), and (b) including the risk factor itself and all identified confounders as independent and the prevalence of lame cows (number of lame cows out of all investigated cows) as dependent variables (adjusted model). Region, herd size, predominant housing system, predominant breed, and season were included in all models. An exception to this was made for the variables of the field cubicle design, where the predominant housing system was not included as a confounder since these variables were assessed only in farms with free stalls with cubicles. Further relevant confounders were included following the respective DAGs. Incidence risk ratios (IRR) were calculated as well, and model diagnostics included visual inspection of residuals for normality and homoscedasticity. Outliers were investigated by Cook’s distance and leverage.

## 3. Results

Six hundred and fifty-nine farms (N: *n* = 240, E: *n* = 247, S: *n* = 172) were included in the analysis of the present study. The median herd size was 96 animals (range 1–2821 cows), and the majority of farms (90.3%) were run conventionally. The predominant breed on most farms was Holstein–Friesian (60.7%), the predominant housing system was loose housing with cubicles (86.8%), followed by mixed (8.2%), straw-based (2.7%), and pasture-based housing systems (2.3%). Mean farm-level lameness prevalence was 30.8% (N: 25.9%, E: 39.4%, S: 25.8%), with a range of lameness prevalence of 0–86.6%. Descriptive statistics of all variables included in the analyses (risk factors and confounders) and their distribution in the three regions are presented in Table 2 and Table 3.

Table 4 displays the results of the univariable analyses of all 24 potential risk factors associated with lameness at the farm level. The adjusted effects of these models and the full list of the considered confounding variables are also presented in Table 4.

### 3.1. Cubicle Design

Cows on farms with predominantly deep cubicles were at the lowest risk of becoming lame compared to raised cubicles (IRR = 1.29) and other cubicle types (IRR = 1.22). The use of bedding material in raised cubicles resulted in a lower risk for lameness compared to raised cubicles without bedding (IRR = 1.14), but the lowest lameness risk was still in deep cubicles (IRR = 0.85). Furthermore, increasing cubicle width was associated with a reduced risk for lameness (IRR = 0.98).

### 3.2. Feeding Management

In farms feeding a total mixed ration (TMR), the risk of being lame was lower than in farms feeding a partial mixed ration or single components (IRR = 0.88).

### 3.3. Claw Health Management, Lameness Assessment, Stocking Density, Floor Design

Further potential risk factors addressing claw health management, lameness assessment, stocking density and floor design did not show significant associations in the adjusted models, although they had significant results in the univariable analyses.

### 3.4. First Lactation Cows

The results of the univariable and the adjusted models for first lactation cows, as well as the confounders included, are presented in Table 5. Similar to the results of all cows (primi- and multiparous cows), feeding a TMR, deep cubicles, and bedded cubicles were factors with a lower risk for first lactation cows to be lame. Different from the model with all cows, the model for first-lactation cows indicates that the lameness of first-lactation cows was influenced by the way lameness assessment was performed. First, lactation cows on farms where lameness assessment was performed in the context of other activities on a regular basis (e.g., milking) had a lower risk for lameness (IRR = 0.69) compared to farms where lameness assessment was a separate work task. The risk of being lame was higher for first lactation cows when the frequency of lameness assessment was less than daily or never compared to daily lameness assessment (IRR = 0.82). Claw trimming management factors, stocking density and floor design did not show significant associations in the adjusted models. This section may be divided by subheadings. It should provide a concise and precise description of the experimental results, their interpretation, and the experimental conclusions that can be drawn.

## 4. Discussion

The present study revealed the most important management-related risk factors for lameness in German dairy cows, for the entire herd and first lactation cows separately.

### 4.1. Cubicle Design

Predominant cubicle type. Predominant cubicle type was strongly associated with lameness in the current study. Deep-bedded cubicles provide more comfort [37,39], especially for the large breed Holstein-Friesian, which is predominantly seen on German dairy farms. Due to better comfort, the overall lying time of the cows increases while the time standing in stalls or in walking alleys decreases [40]. To this end, the pressure exerted on the digits by the body load decreases, which is beneficial for claw health. In particular, lame cows were shown to profit from increased lying times because it helps them to recover and prevents deterioration of their lameness [41,42]. Shorter lying times may be one explanation for the higher lameness prevalence rates on farms with predominantly raised cubicles, just like in our results and the results of other authors [43,44]. Hock injuries, which are more common in raised (rubber mat) cubicles than in deep-bedded cubicles [6], might be considered as another explanation since they have been associated with higher odds of lameness recently [16,37].

Cubicle bedding. Our data showed that the application of bedding material in cubicles reduces lameness prevalence, emphasising the importance of cow comfort. This finding is in accordance with the observations by other authors who reported an association between deep bedding and decreased lameness prevalence [19,45]. Furthermore, our results indicated that bedding in raised cubicles does provide more comfort than no bedding at all, but apparently not as much as in deep-bedded cubicles. A possible explanation is the limited amount of bedding material often seen in raised cubicles, which does not provide sufficient cushion and traction. The quantity of bedding material has been demonstrated to be of importance: when having the free choice between three different amounts of bedding, all cows in the study of Tucker and Weary [39] preferred the option with the highest amount of bedding material. The cows in the latter setting spent more time lying down and less time standing with only the front legs in the stalls compared to cows in cubicles with less bedding. This behaviour of standing only with the front claws in cubicles has been associated with an increased number of claw horn lesions and, hence, lameness [46]. Prolonged lying times are beneficial for claw health and the recovery of existent lesions, as already discussed before.

Cubicle width. Wide cubicles were associated with a lower risk for lameness in our study; this is in alignment with the results of Sogstad et al. [18], who described a negative influence of narrow cubicles on lameness prevalence. The wider the cubicles, the more comfortable they are, and cows lay down more often [47]. However, wider cubicles can entail disadvantages concerning other health aspects, such as a higher risk for integument alterations [6] or more faecal contamination [48]. These disadvantages of wide cubicles can easily be countered by the application of appropriate stall surface design (providing sufficient cushion) and improved stall maintenance (stall cleaning frequency) to benefit from the advantages of a wider cubicle. In summary, our results underline the importance of adequate cubicle design, deep litter systems and proper cubicle maintenance for the reduction of lameness prevalence in German dairy herds.

### 4.2. Feeding Management

Many of the risk factors showing statistically significant associations between feeding management and lameness prevalence in the univariable models did not deliver corresponding results in the adjusted models (Table 4). This indicates that rather confounding factors, such as predominant housing system, farming type, and access to pasture, influence lameness. This issue was particularly distinct in the models of the ration type: in the univariable model, TMR was associated with higher lameness prevalence, whereas in the adjusted model, the result was opposed, and TMR was associated with lower lameness prevalence compared to the feeding of single components or partial mixed rations. In our experience, the ration type seems to be associated with farm size and, consequently, many other confounding factors. Large farms mostly feed TMR, medium-sized farms often feed a partial mixed ration, whereas very small farms mostly feed single components. In the univariable model, the strong effect of the confounders, such as farm size, housing system and pasture access, concealed the positive aspects of TMR, which were revealed in the adjusted model. A reason that accounts for the higher lameness prevalence on farms with partial mixed rations may be the uneven distribution of concentrate, especially for high-yielding cows. Bolus feeding of concentrates has been associated with a higher risk for subclinical ruminal acidosis (SARA), which can lead to laminitis and cause lameness [26,49,50]. However, it must be noted that feeding management, in general, is very hard to interpret. Next to the mentioned confounding factors, other aspects may have an influence, such as differences in energy and protein content, differences in particle sizes or contamination with moulds in grass silages.

### 4.3. Claw Health Management, Lameness Assessment, Stocking Density, Floor Design

The fact that the association of some characteristics (claw health management, lameness assessment, stocking density, and floor design) with farm-level lameness in the univariable models were not significant in the adjusted models indicates that these factors were only pseudo-associated with lameness because important confounders were not considered.

This might be a reason why so many conflicting results were reported from other studies [51,52,53]. In addition, although routine claw trimming was shown to reduce the incidence of claw lesions and lameness [25], it is important to keep in mind that additional factors contribute to the beneficial effect of claw trimming, i.e., application of appropriate trimming techniques and optimal trimming frequency dependent on specific farm and cow characteristics.

Next to the confounders that were included in most models (i.e., region, herd size, predominant housing system, and predominant breed) were farming type and access to pasture/exercise area, which proved to be powerful confounding factors. In our opinion, however, pasture access and farming type must be considered as substitutes for many other management-related variables such as feeding technology, ration design and milking practices, a different mindset of the farmer, lower milk yield, and other breeds, which actually evoke the associations found in the context with pasture access or organic farming.

### 4.4. First Lactation Cows (Lameness Assessment)

Most farms in this study performed lameness monitoring in context with other activities and as a daily task (Table 2); this is consistent with the results of Cutler et al. [54]. Unexpectedly, farms that performed lameness assessment in context with other routine activities, such as feeding or moving cows to or from the milking parlour, showed lower lameness prevalence for first lactation cows compared to farms that implemented a separate work task for lameness monitoring. If lameness monitoring is implemented as a separate work task, it is eventually not performed daily and thus, lame cows are detected later. In addition, cause and effect might be reversed: farms with a high prevalence of lame cows try to encounter the problem by implementing a separate work step for lameness detection. Detected lameness cases must be treated adequately and at an early stage, a consequence which requires well-trained staff and time capacities for the treatment of lame cows.

Our results concerning the method and frequency of lameness assessment for multiparous and first-lactation cows vary. Multiparous cows suffer more frequently from chronic claw disorders than the younger ones [18,55,56]. We assume that farmers were aware of those older cows, which are frequently lame. Lameness assessment might be easier because farmers know which cows they have to monitor, compared to the lameness assessment in first lactation cows, which farmers do not yet know as well. Here, intense and more conscious lameness monitoring is needed to identify new lameness cases.

### 4.5. Farm-Level Lameness Prevalence

The mean farm-level lameness prevalence of 30.8% of the study population was comparable with recently reported prevalences worldwide, which range between 4% and 38% [8,57,58,59]. However, the estimated prevalence of the current study is alarmingly high and unacceptable in the future.

Latest studies conducted in Germany reported markedly lower lameness prevalence rates of 18% [60] and 16% [6]. One possible reason might be the region and, closely related to this, the herd size. The mean number of lactating cows per farm was 106 in Northern Germany and 53 in Southern Germany, whereas in Eastern Germany (a region that was included in the current study but not in the aforementioned German studies), the mean number of lactating cows per farm was 350. Increasing herd size has been associated with increasing lameness prevalence before [61,62,63]. Herd size and milk yield, however, cannot be considered per se as a risk for lameness in dairy cows, as was demonstrated by Cook et al. [64].

### 4.6. Study Design and Limitations

The study population of the current study represented many types of dairy cow farming in Germany and delivered, by including a huge number of farms, in our opinion, reliable results which can be extrapolated on many different housing and management structures. The large sample size allowed a statistical evaluation on the farm level, giving us the possibility to provide results which can be used by farmers to perform reasonable structural changes on their farms to lower the prevalence of lame cows. Bias due to season and year was avoided as much as possible by visiting farms during all seasons and during a period of three years.

Although the selection method of farms was random, the definite decision to participate was voluntary, so the study population cannot be regarded as fully representative. This may have biased results of this study: when especially pro-active farmers with better-managed farms followed the invitation, this could have led to underestimated lameness prevalences. On the other hand, prevalence could also be overestimated if primarily farmers who had concerns about their herd’s health and searched for support participated. Furthermore, it must be noted that the cross-sectional character of this study provided evidence for important associations but did not imply causation. The findings of our study, however, are in accordance with the results of other studies and support the hypotheses of many of our causal diagrams, which were drawn based on biological reasoning.

## 5. Conclusions

Our results reveal the most crucial risk factors for dairy cow lameness on the farm level. Farmers who are willing to improve the situation might check on adequate cubicle width, sufficient bedding material in cubicles/use of deep cubicles, or feeding of a TMR (correctly adapted on requirements), whether these characteristics are already optimised or can be optimised accordingly. As many of these factors are related to cow comfort, especially comfort when lying down, more emphasis is needed to reduce the risk of lame cows in the future. As advisory activities, however, have to be tailor-made for individual farms, distinct farm characteristics have to be considered in veterinary herd health management.

Furthermore, our results provided evidence for the importance of lameness assessment in first-lactation cows. Early detection and prompt and adequate treatment by a qualified claw trimmer of lame first lactation cows can prevent chronic cases of lameness in both primiparous and multiparous cows. In this way, farm-level lameness prevalence can and must be decreased in the long term.

## Figures and Tables

**Table 1:** The number of cows for the herd size categories is small, medium, and large for each region.

Region	Small	Medium	Large
North	1–64	65–113	≥114
East	1–160	161–373	≥374
South	1–29	30–52	≥53

**Table 2:** Categorical variables (risk factors and confounders), their transformation and distribution in regions North, East and South on 659 German dairy farms.

Variable	Scale/Categories	Frequencies *n* (%)			
		North	East	South	Total
Housing/Farm Management					
predominant housing system ^1^	loose housing with cubicles	210 (87.5)	194 (78.5)	168 (97.7)	572 (86.8)
	pasture based	9 (3.8)	6 (2.4)	.	15 (2.3)
	straw based	6 (2.5)	10 (4.0)	2 (1.2)	18 (2.7)
	mixed	15 (6.3)	37 (15.0)	2 (1.2)	54 (8.2)
farming type	conventional	229 (95.4)	225 (91.1)	141 (82.0)	595 (90.3)
	organic	11 (4.6)	22 (8.9)	31 (18.0)	64 (9.7)
access to pasture	no access to pasture	53 (22.2)	113 (46.1)	113 (65.7)	279 (42.5)
	only for lactating cows	23 (9.6)	5 (2.0)	3 (1.7)	31 (4.7)
	only for dry cows	39 (16.3)	74 (30.2)	13 (7.6)	126 (19.2)
	for all cows	124 (51.9)	53 (21.6)	43 (25.0)	220 (33.5)
access to exercise area ^2^	no access to exercise area	169 (70.4)	144 (59.0)	122 (70.9)	435 (66.3)
	for special groups	56 (23.3)	84 (34.4)	24 (14.0)	164 (25.0)
	for all cows	15 (6.3)	16 (6.6)	26 (15.1)	57 (8.7)
automated milking system (AMS)	no AMS	109 (77.9)	144 (82.8)	112 (81.2)	365 (80.8)
	AMS	31 (22.1)	30 (17.2)	26 (18.8)	87 (19.2)
predominant breed ^3,4^	Holstein-Friesian	195 (81.3)	201 (81.4)	4 (2.3)	400 (60.7)
	Simmental	1 (0.4)	2 (0.8)	120 (69.8)	123 (18.7)
	Swiss Brown	.	1 (0.4)	14 (8.1)	15 (2.3)
	others	44 (18.3)	43 (17.4)	34 (19.8)	121 (18.4)
Lameness Assessment					
Method	during other activities	231 (97.9)	224 (92.2)	165 (98.2)	620 (95.8)
	separate work task	5 (2.1)	19 (7.8)	3 (1.8)	27 (4.2)
Frequency	daily	217 (90.4)	210 (85.0)	163 (95.3)	590 (89.7)
	less than daily or never	23 (9.6)	37 (15.0)	8 (4.7)	68 (10.3)
Claw Health Management					
veterinary herd health management program (VHHMP)	no	198 (82.5)	163 (66.3)	169 (99.4)	530 (80.8)
	yes	42 (17.5)	83 (33.7)	1 (0.6)	126 (19.2)
foot bath	no	128 (53.6)	48 (19.4)	164 (96.5)	340 (51.8)
	yes	111 (46.4)	199 (80.6)	6 (3.5)	316 (48.2)
who performs claw trimming	farmer	51 (21.3)	28 (11.3)	70 (41.4)	149 (22.7)
	professional claw trimmer	189 (78.8)	219 (88.7)	99 (58.6)	507 (77.3)
claw trimming frequency	≤1/year	57 (24.1)	22 (9.0)	59 (35.8)	138 (32.1)
	2×/year	140 (59.1)	131 (53.5)	96 (59.2)	367 (35.7)
	≥3×/year	40 (16.9)	92 (37.6)	10 (6.1)	142 (21.9)
claw trimming	in herds/groups and as needed	155 (64.6)	191 (77.3)	64 (37.9)	410 (62.5)
	only in herds/groups or as needed	85 (35.4)	56 (22.7)	105 (62.1)	246 (37.5)
Feeding Management					
feed submission ^5^	<4 times per day	231 (96.3)	222 (89.9)	162 (94.2)	615 (93.3)
	≥4 times per day	9 (3.8)	25 (10.1)	10 (5.8)	44 (6.7)
ration type ^6,7^	total mixed ration	70 (38.9)	189 (81.1)	24 (17.8)	283 (51.6)
	partial mixed ration	46 (25.6)	19 (8.2)	28 (20.7)	93 (17.0)
	single components	64 (35.6)	25 (10.7)	83 (61.5)	172 (31.4)
Stocking Density					
animal: stall ratio (ASR) ^8,1^	>1.2 = poor	20 (8.7)	18 (7.5)	18 (10.5)	56 (8.7)
	1–1.2= moderate	86 (37.4)	50 (20.7)	55 (32.0)	191 (29.7)
	1:1 = good	124 (53.9)	173 (71.8)	99 (57.6)	396 (61.6)
Cubicle Design					
predominant cubicle type ^9^	raised cubicle	90 (46.9)	132 (59.2)	52 (35.4)	274 (48.8)
	deep cubicle	46 (24.0)	27 (12.1)	72 (49.0)	145 (25.8)
	raised deep cubicle	25 (13.0)	49 (22.0)	4 (2.7)	78 (13.9)
	others	31 (16.1)	15 (6.7)	19 (12.9)	65 (11.6)
cubicle type + bedding	raised cubicle without bedding	42 (22.6)	60 (26.9)	46 (30.9)	148 (26.5)
	raised cubicle with bedding	41 (22.0)	67 (30.0)	18 (12.1)	126 (22.6)
	deep cubicle	103 (55.4)	96 (43.0)	85 (57.0)	284 (50.9)
cubicle bedding ^6^	yes	154 (78.6)	165 (73.7)	107 (69.9)	426 (74.3)
	no	42 (21.4)	59 (26.3)	46 (30.1)	147 (25.7)
Floor					
predominant flooring type ^6^	predominant solid/concrete floors	20 (9.0)	111 (48.9)	44 (26.0)	175 (28.4)
	predominant slatted floors	170 (76.9)	66 (29.1)	104 (61.5)	340 (55.1)
	various floors	31 (14.0)	50 (22.0)	21 (12.4)	102 (16.5)
rubber flooring ^6^	yes	25 (11.3)	73 (32.3)	44 (26.0)	142 (23.1)
	no	196 (88.7)	153 (67.7)	125 (74.0)	474 (76.9)
manure removing system ^6^	manure scraper	41 (22.8)	103 (47.7)	50 (32.7)	194 (35.3)
	robot	24 (13.3)	10 (4.6)	11 (7.2)	45 (8.2)
	person	70 (38.9)	83 (38.4)	60 (39.2)	213 (38.8)
	no removing system	45 (25.0)	20 (9.3)	32 (20.9)	97 (17.7)
floor contamination ^6^	clean or single cow pats	40 (18.3)	51 (22.5)	29 (17.3)	120 (19.6)
	<50% contaminated	108 (49.5)	109 (48.0)	77 (45.8)	294 (48.0)
	>50% contaminated or entirely covered with faeces	70 (32.1)	67 (29.5)	62 (36.9)	199 (32.5)
slip resistance on concrete floors ^6^	little resistance	40 (18.1)	63 (27.8)	19 (11.3)	122 (19.8)
	moderate resistance	100 (45.2)	111 (48.9)	77 (45.8)	288 (46.8)
	much resistance	81 (36.7)	53 (23.3)	72 (42.9)	206 (33.4)

N: number of farms; ^1^ >80% of the cows kept in the respective housing system on the day of the farm visit; farms with more than 10% of cows kept in tie-stalls on the day of the farm visit were excluded from the analyses. ^2^ combinations of access to pasture and exercise area. ^3^ categorised. ^4^ >80% of the cows were assigned to the respective breed on the day of the farm visit. ^5^ mean, dichotomised ration for lactating cows. ^6^ mode. ^7^ ration for lactating and dry cows. ^8^ weighted per compartment, mean, categorised. ^9^ >80% of the cows were kept in a loose housing system with the according cubicle type on the day of the farm visit.

**Table 3:** Continuous variables (risk factors and confounders) and their distribution on 659 German dairy farms.

Region	N	Mean	Min	0.25	Median	0.75	Max
Housing/Farm Management							
herd size							
North	240	106.65	10	61	90	129	991
East	247	350.47	1	136	251	449	2821
South	172	53.58	7	34	51	65	231
proportion of first lactation cows in %							
North	231	29.31	6.25	24.05	29.70	33.78	57.69
East	244	29.76	9.68	25.89	29.84	33.33	66.60
South	160	28.77	5.88	24.77	28.90	33.63	53.48
culling rate due to lameness in % (log10)							
North	236	0.52	0.00	0.00	0.58	0.83	1.45
East	231	0.60	0.00	0.35	0.64	0.89	1.50
South	171	0.29	0.00	0.00	0.00	0.64	1.34
Extraversion of the farmer ^1^							
North	173	3.79	2.0	3.5	4.0	4.0	5.0
East	167	3.73	2.0	3.5	4.0	4.0	5.0
South	146	3.80	2.0	3.5	4.0	4.0	5.0
Openness of the farmer ^1^							
North	173	3.46	2.0	3.0	3.5	4.0	5.0
East	167	3.52	2.0	3.0	3.5	4.0	5.0
South	146	3.42	1.5	3.0	3.5	4.0	5.0
Feeding Management							
roughage ratio in % dry matter							
North	156	64.87	38.39	56.33	64.36	72.88	93.17
East	199	70.21	26.19	63.09	70.61	77.35	99.38
South	64	68.52	49.79	61.03	69.03	74.50	96.17
Stocking Density							
max. animals per compartment (log)							
North	240	1.85	1.08	1.72	1.85	2.00	2.42
East	246	1.93	0.30	1.72	1.96	2.11	2.62
South	172	1.63	0.85	1.51	1.66	1.76	2.24
animal: feeding place ratio (AFR) ^2^							
North	231	1.18	0.25	0.96	1.10	1.36	3.07
East	241	1.31	0.51	1.03	1.26	1.51	7.50
South	172	1.06	0.57	0.92	1.00	1.18	2.26
Cubicle Design							
cubicle width ^1^ in cm							
North	222	112.14	99	110	113	115	121
East	227	112.45	101	110	113	114	139
South	169	115.61	101	113	116	119	126
neck rail to curb distance ^1^ in cm							
North	222	197.49	176	191	198	204	222
East	227	195.30	168	189	195	200	238
South	169	194.55	170	189	194	200	221
brisket board height ^1^ in cm							
North	223	12.91	0	0	15	19	41
East	228	9.85	0	0	3	19	37
South	169	12.03	0	6	12	18	40
curb height ^1^ in cm							
North	222	23.24	6	21	23	25	39
East	227	22.97	8	20	23	26	43
South	168	20.91	4	18	21	23	35

N: number of farms. Min: minimum. Max: maximum. ^1^ median. ^2^ weighted per compartment.

**Table 4:** Results of the univariable analyses and the adjusted models of potential risk factors associated with the percentage of lame cows per farm on 659 German dairy farms.

		Not Adjusted Univariable Analysis			Adjusted Model					
Risk Factor		Crude Estimate	OR	*p*-Value	Adjusted Estimate	SE	*p*-Value	IRR	95% CI	
Cubicle Design										
predominant cubicle type ^1^				0.000			0.000			
	deep cubicle	Reference			Reference					
	raised cubicle	0.36	1.43	0.000	0.25	0.0463	0.000	1.29	1.17	13.01
	raised deep cubicle	0.21	1.23	0.001	0.04	0.0617	0.465	1.04	0.92	8.21
	others	0.19	1.21	0.007	0.20	0.0656	0.002	1.22	1.07	11.68
cubicle ^2^				0.000			0.000			
	raised cubicle with bedding	Reference			Reference					
	raised cubicle without bedding	0.09	1.09	0.134	0.14	0.0505	0.006	1.14	1.04	9.92
	deep cubicle	−0.23	0.80	0.000	−0.17	0.0450	0.000	0.85	0.77	5.50
cubicle bedding ^1^										
	yes	Reference	.		Reference		.			
	no	0.24	1.27	0.000	0.15	0.0470	0.002	1.16	1.05	10.11
cubicle width in cm ^3^		−0.02	0.98	0.000	−0.02	0.0045	0.000	0.98	0.97	6.90
neck rail to curb distance in cm ^4^		0.00	1.00	0.249	0.00	0.0020	0.751	1.00	1.00	7.12
brisket board height in cm ^5^		0.00	1.00	0.047	0.00	0.0018	0.415	1.00	0.99	7.09
curb height in cm ^6^		0.00	1.00	0.422	0.00	0.0038	0.924	1.00	0.99	7.13
Feeding Management										
feed submission ^7,8^										
	≥4 times per day	Reference			Reference					
	<4 times per day	−0.10	0.90	0.193	−0.01	0.0770	0.858	0.99	0.85	7.48
ration type ^7,9^				0.000			0.079			
	upgraded mixed ration	Reference			Reference		.	.	.	.
	total mixed ration	0.15	1.16	0.011	−0.13	0.0566	0.025	0.88	0.79	5.94
	single components	−0.24	0.79	0.001	−0.06	0.0615	0.307	0.94	0.84	6.76
roughage ratio in % dry matter ^7,10^		0.00	1.00	0.294	0.00	0.0019	0.412	1.00	0.99	7.09
Claw Health Management										
who performs claw trimming ^7,11^										
	professional claw trimmer	Reference			Reference		.	.	.	.
	farmer	−0.18	0.83	0.000	−0.10	0.0538	0.063	0.91	0.82	6.26
claw trimming frequency ^7,12^				0.000			0.417			
	2×/year	Reference			Reference					
	≥3×/year	0.18	1.20	0.000	0.00	0.0543	0.976	1.00	0.90	7.52
	≤1/year	−0.31	0.73	0.000	−0.08	0.0629	0.190	0.92	0.81	6.45
foot bath ^7,13^										
	yes	Reference			Reference					
	no	−0.35	0.70	0.000	−0.07	0.0477	0.166	0.94	0.85	6.56
Lameness Assessment										
method ^7,14^										
	seperate work task	Reference			Reference					
	during other activities	−0.30	0.74	0.003	−0.17	0.1078	0.106	0.84	0.68	5.78
frequency ^7,15^										
	less than daily or never	Reference			Reference					
	daily	−0.26	0.77	0.000	−0.11	0.0681	0.103	0.89	0.78	6.18
Stocking Density										
max. animals per compartement ^7,16^		0.69	2.00	0.000	0.07	0.0990	0.452	1.08	0.89	9.12
ASR7 ^17^				0.003			0.061			
	good	Reference			Reference					
	moderate	−0.15	0.86	0.001	−0.08	0.0417	0.072	0.93	0.85	6.42
	poor	0.02	1.02	0.794	0.07	0.0682	0.312	1.07	0.94	8.74
AFR ^7,18^		0.14	1.15	0.007	−0.02	0.0468	0.676	0.98	0.89	7.16
Floor Design										
predominant flooring type ^7,19^				0.001			0.235			
	predominant solid/concrete floors	Reference			Reference		.	.	.	.
	predominant slatted floors	−0.16	0.85	0.000	0.08	0.0458	0.089	1.08	0.99	8.71
	various floors	−0.03	0.97	0.645	0.04	0.0549	0.471	1.04	0.93	8.12
rubber flooring ^7,20^										
	yes	Reference			Reference					
	no	−0.04	0.96	0.443	0.05	0.0441	0.226	1.05	0.97	8.26
manure removing system ^7,21^				0.009			0.344			
	manure scraper	Reference			Reference					
	robot	−0.08	0.93	0.345	0.02	0.0878	0.796	1.02	0.86	8.11
	person	−0.07	0.93	0.135	−0.03	0.0573	0.612	0.97	0.87	7.11
	no removing system	−0.21	0.81	0.001	−0.10	0.0733	0.157	0.90	0.78	6.30

OR: Odds ratio; IRR: incidence risk ratio; 95% CI: 95% confidence interval; ASR: animal-stall ratio; AFR: animal-feeding place ratio; VHHMP: veterinary herd health management program. ^1^ adjusted for region, herd size, predominant breed, season, use of VHHMP. ^2^ adjusted for region, herd size, predominant breed, and season. ^3^ adjusted for region, herd size, predominant breed, season, predominant cubicle type, proportion of first lactation cows, and ASR. ^4^ adjusted for region, herd size, predominant breed, season, access to pasture/exercise area, predominant cubicle type, cubicle bedding, proportion of first lactation cows, and use of VHHMP. ^5^ adjusted for region, herd size, predominant breed, season, access to pasture/exercise area, predominant cubicle type, cubicle width, neck rail to curb distance, the proportion of first lactation cows, and use of VHHMP. ^6^ adjusted for region, herd size, predominant breed, season, access to pasture/exercise area, predominant cubicle type, cubicle width, predominant flooring type, rubber flooring, floor contamination, and slip resistance on concrete floors. ^7^ adjusted for region, herd size, predominant housing system, predominant breed, and season. ^8^ adjusted for farming type, access to pasture/exercise area, predominant flooring type, ration type, and ASR. ^9^ adjusted for farming type. ^10^ adjusted for farming type, access to pasture/exercise area, ration type, and feed submission. ^11^ adjusted for farming type, use of VHHMP, openness, and extraversion. ^12^ adjusted for farming type, access to pasture/exercise area, use of VHHMP, who performs claw trimming, rubber flooring, slip resistance on concrete floors, openness, and extraversion. ^13^ adjusted for farming type, access to pasture/exercise area, use of VHHMP, who performs claw trimming, claw trimming frequency, culling rate due to lameness, proportion first lactation cows. ^14^ adjusted for max. animals per compartment, who performs claw trimming, use of VHHMP, openness, and extraversion. ^15^ adjusted for max. animals per compartment, who performs claw trimming, and method of lameness assessment. ^16^ adjusted for access to pasture/exercise area, predominant flooring type, rubber flooring, and use of VHHMP. ^17^ adjusted for access to pasture/exercise area, max. animals per compartment, predominant flooring type, rubber flooring, use of VHHMP. ^18^ adjusted for access to the pasture/exercise area milking system. ^19^ adjusted for access to pasture/exercise area, and rubber flooring. ^20^ adjusted for predominant flooring type use of VHHMP. ^21^ adjusted for predominant flooring type and max. animals per compartment.

**Table 5:** Results of the univariable analyses and the adjusted models of potential risk factors associated with the percentage of lame first lactation cows per farm on 659 German dairy farms.

		Not Adjusted Univariable Analysis			Adjusted Model					
Risk Factor		Crude Estimate	OR	*p*-Value	Adjusted Estimate	SE	*p*-Value	IRR	95% CI	
Cubicle Design										
predominant cubicle type ^1^				0.000			0.000			
	deep cubicle	Reference			Reference					
	raised cubicle	0.37	1.45	0.000	0.30	0.07	0.000	1.35	1.17	15.00
	raised deep cubicle	0.11	1.11	0.224	0.01	0.09	0.932	1.01	0.84	7.89
	others	0.27	1.31	0.007	0.27	0.10	0.006	1.31	1.08	14.49
Cubicle ^2^				0.000			0.000			
	raised cubicle with bedding	Reference			Reference					
	raised cubicle without bedding	0.18	1.20	0.012	0.22	0.07	0.002	1.25	1.09	12.45
	deep cubicle	−0.22	0.81	0.001	−0.18	0.07	0.005	0.83	0.73	5.46
cubicle bedding ^1^										
	yes	Reference			Reference					
	no	0.33	1.40	0.000	0.22	0.07	0.001	1.24	1.09	12.23
cubicle width in cm ^3^		−0.03	0.97	0.000	−0.02	0.01	0.001	0.98	0.96	6.83
neck rail to curb distance in cm ^4^		0.00	1.00	0.768	0.00	0.00	0.682	1.00	0.99	7.10
brisket board height in cm ^5^		0.00	1.00	0.172	0.00	0.00	0.600	1.00	1.00	7.14
curb height in cm ^6^		−0.01	0.99	0.128	0.00	0.01	0.767	1.00	0.99	7.12
Feeding Management										
feed submission ^7,8^										
	≥4 times per day	Reference			Reference					
	<4 times per day	−0.19	0.82	0.054	−0.10	0.11	0.365	0.91	0.73	6.59
ration type ^7,9^				0.008			0.055			
	upgraded mixed ration	Reference			Reference					
	total mixed ration	0.05	1.05	0.543	−0.19	0.09	0.024	0.82	0.70	5.48
	single components	−0.18	0.83	0.056	−0.04	0.10	0.649	0.96	0.79	7.19
roughage ratio in % dry matter ^7,10^		0.00	1.00	0.893	0.00	0.00	0.254	1.00	0.99	7.07
Claw Health Management										
who performs claw trimming ^7,11^										
	professional claw trimmer	Reference			Reference					
	farmer	−0.16	0.85	0.025	−0.12	0.09	0.168	0.89	0.75	6.21
claw trimming frequency ^7,12^				0.000			0.437			
	2×/year	Reference			Reference					
	≥3×/year	0.16	1.18	0.009	−0.01	0.08	0.871	0.99	0.84	7.47
	≤1/year	−0.22	0.80	0.005	−0.13	0.11	0.199	0.88	0.71	6.23
foot bath ^7,13^										
	yes	Reference	.		Reference					
	no	−0.25	0.78	0.000	−0.03	0.07	0.637	0.97	0.84	7.15
Lameness Assessment										
method ^7,14^										
	seperate work task	Reference			Reference					
	during other activities	−0.40	0.67	0.001	−0.38	0.14	0.008	0.68	0.52	4.41
frequency ^7,15^										
	less than daily or never	Reference			Reference					
	daily	−0.34	0.71	0.000	−0.21	0.90	0.022	0.81	0.14	12.12
Stocking Density										
max. animals per compartement ^7,16^		0.53	1.71	0.000	0.12	0.15	0.422	1.13	0.84	10.50
ASR7 ^17^				0.111			0.401			
	good	Reference			Reference					
	moderate	−0.12	0.89	0.043	−0.05	0.06	0.486	0.95	0.84	6.92
	poor	0.02	1.02	0.841	0.09	0.10	0.357	1.10	0.90	9.49
AFR ^7,18^		0.08	1.08	0.219	−0.01	0.06	0.837	0.99	0.87	7.38
Floor Design										
predominant flooring type ^7,19^				0.047			0.669			
	predominant solid/concrete floors	Reference			Reference					
	predominant slatted floors	−0.13	0.88	0.029	0.05	0.07	0.447	1.05	0.92	8.45
	various floors	0.00	1.00	0.981	0.05	0.08	0.500	1.05	0.91	8.51
rubber flooring ^7,20^										
	yes	Reference			Reference					
	no	0.02	1.02	0.804	0.02	0.06	0.804	1.02	0.90	7.80
manure removing system ^7,21^				0.029			0.206			
	manure scraper	Reference			Reference					
	robot	−0.02	0.98	0.871	0.09	0.13	0.482	1.09	0.85	9.72
	person	−0.02	0.98	0.777	0.03	0.08	0.675	1.03	0.88	8.24
	no removing system	−0.24	0.78	0.004	−0.13	0.11	0.238	0.88	0.71	6.26

OR: Odds ratio; IRR: incidence risk ratio; 95% CI: 95% confidence interval; ASR: animal-stall ratio; AFR: animal-feeding place ratio; VHHMP: veterinary herd health management program. ^1^ adjusted for region, herd size, predominant breed, season, and use of VHHMP. ^2^ adjusted for region, herd size, predominant breed, and season. ^3^ adjusted for region, herd size, predominant breed, season, predominant cubicle type, proportion of first lactation cows, and ASR. ^4^ adjusted for region, herd size, predominant breed, season, access to pasture/exercise area, predominant cubicle type, cubicle bedding, proportion of first lactation cows, and use of VHHMP. ^5^ adjusted for region, herd size, predominant breed, season, access to pasture/exercise area, predominant cubicle type, cubicle width, neck rail to curb distance, the proportion of first lactation cows, and use of VHHMP. ^6^ adjusted for region, herd size, predominant breed, season, access to pasture/exercise area, predominant cubicle type, cubicle width, predominant flooring type, rubber flooring, floor contamination, and slip resistance on concrete floors. ^7^ adjusted for region, herd size, predominant housing system, predominant breed, and season. ^8^ adjusted for farming type, access to pasture/exercise area, predominant flooring type, ration type, and ASR. ^9^ adjusted for farming type. ^10^ adjusted for farming type, access to pasture/exercise area, ration type, and feed submission. ^11^ adjusted for farming type, use of VHHMP, openness, and extraversion. ^12^ adjusted for farming type, access to pasture/exercise area, use of VHHMP, who performs claw trimming, rubber flooring, slip resistance on concrete floors, openness, and extraversion. ^13^ adjusted for farming type, access to pasture/exercise area, use of VHHMP, who performs claw trimming, claw trimming frequency, culling rate due to lameness, and proportion first lactation cows. ^14^ adjusted for max. animals per compartment, who performs claw trimming, use of VHHMP, openness, and extraversion. ^15^ adjusted for max. animals per compartment, who performs claw trimming, and method of lameness assessment. ^16^ adjusted for access to pasture/exercise area, predominant flooring type, rubber flooring, and use of VHHMP. ^17^ adjusted for access to pasture/exercise area, max. animals per compartment, predominant flooring type, rubber flooring, and use of VHHMP. ^18^ adjusted for access to pasture/exercise area, and milking system. ^19^ adjusted for access to pasture/exercise area, and rubber flooring. ^20^ adjusted for predominant flooring type and use of VHHMP. ^21^ adjusted for predominant flooring type, max. animals per compartment.

## Data Availability

The datasets presented in this article are not readily available because the data were acquired through cooperation between different universities. These universities signed a cooperation contract and agreed that any data transfer to interested persons is not allowed without an additional formal contract. Data are available for qualified researchers who sign a contract with the project consortium. This contract will include guarantees of the obligation to maintain data confidentiality in accordance with the provisions of German data protection law. Currently, there exists no data access committee nor another body that could be contacted for the data; a committee will be founded for this purpose. This future committee will consist of the authors as well as members of the related universities. Requests to access the datasets should be directed to: A. Campe, Institute of Biometry, Epidemiology and Information Processing at the University of Veterinary Medicine, Hannover, Bünteweg 2, 30559 Hannover, Germany, Email: anely.campe@tiho-hannover.de, M. Hoedemaker, Clinic for Cattle at the University of Veterinary Medicine, Hannover, Bischofsholer Damm 15, 30173 Hannover, Germany, Email: martina.hoedemaker@tiho-hannover.de, R. Merle, Institute of Veterinary Epidemiology and Biostatics at the School of Veterinary Medicine at the Free University Berlin, Königsweg 67, 14163 Berlin, Germany, Email: roswitha.merle@fu-berlin.de.
